# Enhanced Blood Pressure–Lowering Effect of Olmesartan in Hypertensive Patients With Chronic Kidney Disease–Associated Sympathetic Hyperactivity: HONEST Study

**DOI:** 10.1111/jch.12132

**Published:** 2013-05-31

**Authors:** Kazuomi Kario, Ikuo Saito, Toshio Kushiro, Satoshi Teramukai, Yoshihiro Mori, Katsutoshi Hiramatsu, Fumiaki Kobayashi, Kazuyuki Shimada

**Affiliations:** 1 Division of Cardiovascular Medicine, Department of Medicine, Jichi Medical University School of MedicineShimotsuke, Japan; 2 Keio University Health CenterYokohama, Japan; 3 Health Planning Center, Nihon University School of MedicineTokyo, Japan; 4 Innovative Clinical Research Center, Kanazawa UniversityKanazawa, Japan; 5 Daiichi Sankyo Co., LtdTokyo, Japan; 6 Shin-Oyama City HospitalOyama, Japan

## Abstract

To investigate the blood pressure (BP)–lowering effect of olmesartan in relation to chronic kidney disease (CKD)–associated sympathetic nerve activity, a subanalysis was performed using data from the first 16 weeks of the Home BP Measurement With Olmesartan-Naive Patients to Establish Standard Target Blood Pressure (HONEST) study, a prospective observational study of hypertensive patients. Essential hypertensive patients who took no antihypertensive agent at baseline were classified based on baseline morning home systolic BP (MHSBP) in quartiles. In each class, patients were further classified based on baseline morning home pulse rate (MHPR). A subgroup analysis in patients with/without chronic kidney disease (CKD) was performed. A total of 5458 patients (mean age, 63.0 years; 51.6% women) were included. In the 4th quartile of baseline MHSBP (≥165 mm Hg), patients with MHPR ≥70 beats per minute had a greater BP reduction (by 3.2 mm Hg) than those with MHPR <70 beats per minute after 16 weeks of olmesartan-based treatment (*P*=.0005). An even greater BP reduction (by 6.6 mm Hg) was observed in patients with CKD than in patients without CKD in this group (*P*=.0084). Olmesartan was more effective in hypertensive patients with high MHSBP and MHPR ≥70 beats per minute, especially in patients with CKD. Olmesartan may have enhanced BP-lowering effects by improving renal ischemia in hypertensive CKD patients with potential increased sympathetic nerve activity.

Hypertensive patients commonly have increased sympathetic nerve activity.^1^ Morning hypertension and morning surge in blood pressure (BP) are particularly known to be caused by increased sympathetic nerve activity as well as lack of sustained effects of antihypertensive drugs.^2^,^3^ A marked increase in sympathetic nerve activity has been reported at the time of awakening.^5^ In addition, heart rate, which is associated with prognosis of hypertensive patients, is also known as an indicator of increased sympathetic nerve activity.^6^–^7^ Therefore, compared with clinic BP and pulse rate (PR), morning home BP and PR are thought to reflect the status of sympathetic nerve activity more accurately.

Moreover, patients with conditions such as obesity leading to the metabolic syndrome and chronic kidney disease (CKD), who have a marked increase in sympathetic nerve activity, often have concomitant morning hypertension.^8^–^11^ Particularly in patients with CKD, increased sympathetic nerve activity is observed from the early stages and becomes more prominent according to the disease progression.^12^ Two mechanisms are thought to be involved in the activation of renal sympathetic nerves in patients with CKD. The first mechanism is that mild ischemia of renal tissues activates the hypothalamic area and the sympathetic nervous system center in the medulla oblongata through the afferent nerve pathway. The second one is that ischemia of renal tissues activates the renin-angiotensin system, and the resultant angiotensin II (Ang II) activates the central sympathetic nervous system.^13^

Consequently, we proposed the following hypothesis: In hypertensive patients with high PR (indicating increased sympathetic nerve activity), angiotensin receptor blockers (ARBs) yield a potent BP-lowering effect through suppression of sympathetic nerve activity. Especially in hypertensive patients with concomitant CKD who are characterized by increased sympathetic nerve activity, an even greater BP reduction can be obtained. In order to verify this hypothesis, we conducted the present analysis using data from a large-scale observational study of an ARB, olmesartan. The Home BP Measurement With Olmesartan-Naive Patients to Establish Standard Target Blood Pressure (HONEST) study is a prospective observational study following >20,000 patients receiving olmesartan-based antihypertensive treatment for 2 years. The time from start of treatment to first occurrence of cardiovascular events is the primary endpoint.^14^

Here, we evaluated patients untreated with antihypertensive drugs using data as of 16 weeks from the HONEST study in order to test our hypothesis. Morning home BP was used to evaluate the BP-lowering effect, and morning home PR (MHPR) was used to evaluate effects on sympathetic nerve activity.

## Methods

The aims and protocol of the HONEST study have already been reported.^14^ This study was a large-scale prospective observational study with a 2-year follow-up by September 30, 2012. The study was registered at http://www.umin.ac.jp/ctr/index.htm with the unique trial number UMIN000002567. The study protocol was approved by the Ethical Committee of Daiichi Sankyo Co., Ltd., and it conformed with the pharmaceutical affairs laws of Japan and was approved by the Ministry of Health, Labour and Welfare of Japan before commencement. This study was carried out in medical institutions registered in compliance with Good Post-marketing Study Practice in Japan and internal regulations for clinical studies at each institution.

Briefly, participants were olmesartan-naïve with essential hypertension. Written informed consent was obtained from all patients at the start of the study. Patients were excluded if they had a history of recent cardiovascular events (eg, myocardial infarction, stroke, and cardiovascular interventions), or if cardiovascular interventions were planned. Olmesartan (generally 10 mg/d or 20 mg/d) was administered at each participating physician's discretion. No restriction was placed on prior antihypertensive drug treatment, with the exception of prior use of olmesartan, or on the use of combination antihypertensive drug treatment during the study. The data included patient characteristics (eg, disease history and complications), clinic BP and home BP, clinic PR, MHPR, clinical laboratory test values, and the incidence of cardiovascular events and adverse events during the study period. The present analysis used the first 16 weeks of data from the HONEST study for patients who received olmesartan and took no antihypertensive agent before the study, avoiding the influence of prior antihypertensive drug treatment.

### Home BP Measurements

Patients who already owned a sphygmomanometer based on the cuff-oscillometric principal were registered, but electrical devices for home BP measurements were not standardized. All such devices available in Japan have been validated and approved by the Ministry of Health, Labour and Welfare of Japan. At the time of obtaining informed consent, patients were asked to measure home BP twice in the morning and twice at bedtime according to the Japan Society of Hypertension 2009 guidelines,^15^ namely, in the morning (within 1 hour after waking up, after urination, before dosing in the morning, before breakfast, and 1 or 2 minutes after resting in a sitting position), and at bedtime (after 1 or 2 minutes of resting in a sitting position). We analyzed only the first measurement of home BP and PR in the morning at baseline and at 16 weeks. Home BP at each measurement point is defined as an averaged value over 2 days.

### Definition of Patients With CKD

The patients with CKD at baseline were defined as having an estimated glomerular filtration rate (eGFR) <60 mL/min/1.73 m^2^, or proteinuria ≥2+ on dipstick test, or proteinuria 1+ and renal disease as a complication at study entry, or both. eGFR was calculated by the following formula devised for the Japanese population^16^: eGFR=194×age (years)^−0.287^×SCr^−1.094^ (×0.739 in women), where serum creatinine (SCr) levels measured within 12 months prior to study onset were used.

### Data Analysis

Patients were classified based on baseline morning home systolic BP (MHSBP) in quartiles. Patients were also stratified into high and low MHPR groups using the cut-off value of MHPR 70 beats per minute (bpm) according to previous reports.^17^–^18^ For the comparison of changes in MHSBP and MHPR after 16 weeks of olmesartan-based treatment by quartiles of baseline MHSBP, by baseline MHPR, or by low and high MHPR in quartiles of MHSBP, analysis of variance was performed with adjustments for sex, age, disease duration, diabetes mellitus, smoking status, and alcohol drinking status. For comparison of baseline characteristics between CKD and non-CKD patients, chi-square test was used for categorical variables and *t* test for continuous variables. As to the comparison of BP-lowering effect, age and smoking status were used as adjustment factors. Patients with missing values of BP and PR were excluded from the analyses. Effect modification by CKD, on differences of the changes in MHSBP between high (≥70 bpm) and low (<70 bpm) baseline MHPR, was examined by interaction test between MHPR (high/low) and patients with/without CKD.

All tests were two-sided, and *P*<.05 was considered statistically significant. Continuous variables and categorical variables were expressed as mean±standard deviations. SAS release 9.2 (SAS Institute, Cary, NC) was used for all statistical analyses.

## Results

### Patient Disposition

The subanalysis was conducted in 5458 unmedicated hypertensive patients at baseline with the data of MHSBP and MHPR both at baseline and at 16 weeks after olmesartan administration.

### Patient Background

The baseline characteristics of the patients are presented in the Table1. The mean age of the patients was 63.0 years (range, 16–96 years); 51.6% of patients were women. Of 5458 patients, 891 (16.3%) had concomitant CKD. Compared with patients without CKD (non-CKD), CKD patients had a higher percentage of female patients, an older mean age, longer duration of disease, and higher percentages of patients with a history of cerebrovascular/cardiovascular disease (*P*<.05 for all comparisons). Moreover, in CKD patients, body mass index, percentages of current smokers and regular alcohol drinkers, and morning home and clinic diastolic BP were lower (*P*<.001 for all comparisons). There was no significant difference in the morning home and clinic systolic BP and PR between the two groups.

**Table 1:** Baseline Characteristics

Patients	All Patients (N=5458)^a^	CKD (−) (n=4542)	CKD (+) (n=891)	*P* Value^b^
Women, %	51.6	50.9	55.4	.0127
Age (range), y	63.0±12.0 (16–96)	61.9±11.7 (16–95)	68.9±11.5 (27–96)	<.0001
BMI, kg/m^2^	24.12±3.57	24.22±3.58	23.63±3.46	.0001
Duration of hypertension, y	3.15±4.00	2.96±3.89	4.19±4.45	<.0001
Cerebrovascular disease, %	3.4	3.0	5.1	.0014
Cardiovascular disease, %	1.5	1.2	3.3	<.0001
Dyslipidemia, %	38.6	38.2	40.1	.3005
Diabetes mellitus, %	14.5	14.2	15.8	.1951
Current smoker, %	13.5	14.2	9.5	.0003
Regularly drinks alcohol, %	17.1	18.2	11.6	<.0001
BP parameters				
Morning home SBP, mm Hg	156.2±15.1	156.2±15.0	156.2±15.2	.9658
Morning home DBP, mm Hg	90.8±11.0	91.2±10.9	88.8±11.1	<.0001
Morning home pulse rate, bpm	71.8±9.7	71.8±9.6	71.9±10.0	.6903
Clinic SBP, mm Hg	159.2±17.3	159.3±17.2	159.1±17.6	.6768
Clinic DBP, mm Hg	91.5±12.2	92.0±12.1	89.4±12.5	<.0001
Clinic pulse rate, bpm	74.4±10.8	74.4±10.7	74.4±11.4	.9928

Abbreviations: BMI, body mass index; BP, blood pressure; bpm, beats per minute; CKD, chronic kidney disease; DBP, diastolic blood pressure; SBP, systolic blood pressure. Data are shown as means±standard deviation or number of patients (percentage).

a Including 25 patients with unknown status of CKD.

b CKD(−) vs (+).

### Dose of Olmesartan

Patients visited the hospital several times during the study period, and physicians adjusted the dose of olmesartan by checking for effective BP control. The mean (±standard deviation) dose of olmesartan in all patients, in CKD patients, and in non-CKD patients increased from 16.86±6.00, 17.04±6.45, and 16.83±5.89 mg at baseline to 17.97±6.94, 18.08±7.29, and 17.96±6.85 mg at 16 weeks, respectively.

### Changes in MHSBP and MHPR by Quartiles of Baseline MHSBP

**Figure 1:**
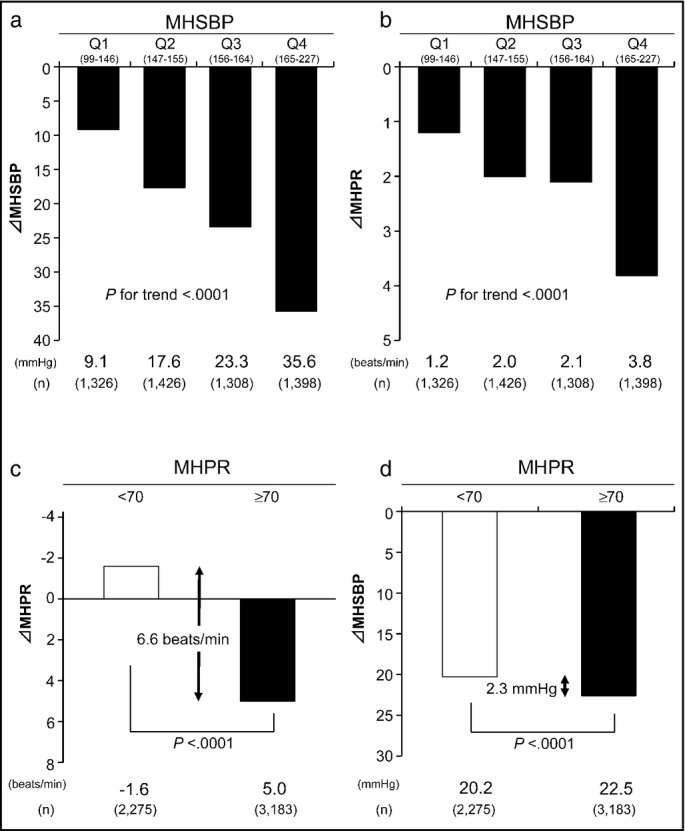
Changes in morning home systolic blood pressure (MHSBP) (a) and morning home pulse rate (MHPR) (b) after 16 weeks of olmesartan treatment classified by MHSBP at baseline and changes in MHPR (c) and MHSBP (d) after 16-week olmesartan treatment classified by MHPR at baseline. ⊿MHSBP (a), ⊿MHPR (b), and ⊿MHSBP (d) are adjusted by sex, age (3 stages), duration of hypertension, diabetes mellitus, smoking, and alcohol, respectively. ⊿MHPR (c) is adjusted by sex, age (3 stages), duration of hypertension, diabetes mellitus, smoking, alcohol, and MHSBP (10 stages).

Figure 1a and 1b show the changes in MHSBP and MHPR after 16 weeks of olmesartan treatment in patients classified into quartiles based on their baseline MHSBP. Significantly greater decreases in MHSBP and MHPR were noted in patients with higher baseline MHSBP (*P*<.0001 for both comparisons). Specifically, the changes from baseline in MHSBP (ΔMHSBP) and MHPR (ΔMHPR) were 35.6 mm Hg and 3.8 bpm, respectively, in the fourth quartile, whereas they were 9.1 mm Hg and 1.2 bpm, respectively, in the first quartile. A similar significant reduction pattern was also observed when the relation was analyzed by percentage reduction. The percentage reduction in each quartile from MHSBP quartile 1 (Q1) to MHSBP quartile 4 (Q4), was 6.4%, 11.6%, 14.6%, and 20.1% for MHSBP (*P*<.0001) and 1.1%, 2.0%, 2.3%, and 4.3% for MHPR (*P*<.0001), respectively.

### Changes in MHSBP and MHPR by Baseline MHPR

Figure 1c and 1d compare the changes in MHSBP and MHPR between patients with high (≥70 bpm) and low (<70 bpm) baseline MHPR after 16 weeks of olmesartan treatment. Compared with the group with low baseline MHPR, the group with high baseline MHPR had significantly greater decreases in MHPR and MHSBP, with the differences being 6.6 bpm and 2.3 mm Hg, respectively (*P*<.0001 for both comparisons). A similar significant reduction pattern was also observed when the relation was analyzed by percentage reduction. The percentage reductions of MHPR <70 bpm and MHPR ≥70 bpm was −2.7% and 6.1% for MHPR (*P*<.0001) and 12.6% and 13.7% for MHSBP (*P*<.0001), respectively.

### Changes in MHSBP by Baseline MHSBP and MHPR

**Figure 2:**
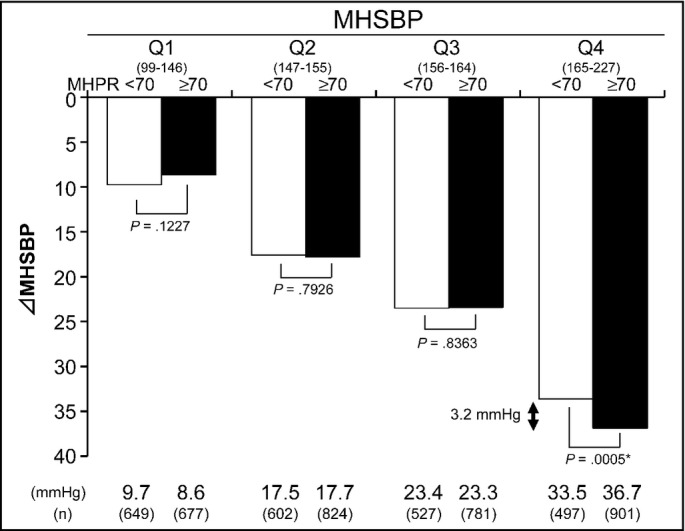
Change in morning home systolic blood pressure (MHSBP) after 16 weeks of olmesartan treatment classified by MHSBP and morning home pulse rate (MHPR) at baseline. ⊿MHSBP is adjusted by sex, age (3 stages), duration of hypertension, diabetes mellitus, smoking, and alcohol. *P<.001: analysis of variance (MHPR <70 vs ≥70 beats per minute).

To exclude the possibility that BP- and PR-lowering effects of olmesartan accounted only for consequence of phenomenon of regression to the mean, we stratified study patients by baseline MHSBP and MHPR, and compared changes in MHSBP after 16 weeks of olmesartan treatment (Figure 2). In the first to third quartile, no significant difference was observed between patients with high and low baseline MHPR. By contrast, in the fourth quartile, patients with high baseline MHPR had a significantly greater reduction in MHSBP by 3.2 mm Hg compared with those with low baseline MHPR (*P*=.0005). A similar pattern of reduction was also observed when the relation was analyzed by percentage reductions from baseline MHSBP (Q1–Q3, not significant; Q4, *P*=.0010).

### Changes in MHSBP by Baseline MHSBP and MHPR in Patients With or Without CKD

**Figure 3:**
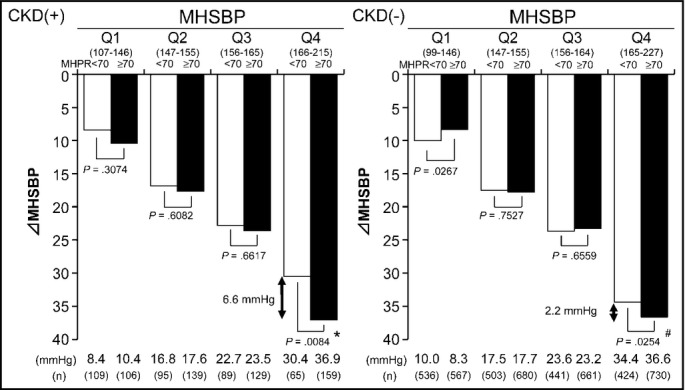
Changes in morning home systolic blood pressure (MHSBP) of patients with chronic kidney disease (CKD) and no CKD after 16-week olmesartan treatment classified by MHSBP and morning home pulse rate (MHPR) at baseline. ⊿MHSBP(CKD) is adjusted by age (3 stages) and smoking and ⊿MHSBP(non-CKD) is adjusted by sex, age (3 stages), duration of hypertension, history of drug allergy, and alcohol. *P<.01: MHPR <70 vs ≥70 beats per minute. #*P*<.05: analysis of variance (MHPR <70 vs ≥70 bpm).

Figure 3 compares the changes in MHSBP after 16 weeks of olmesartan-based treatment in patients classified by baseline MHSBP and MHPR and the presence of concomitant CKD. In the first to third quartile, differences between patients with high and low baseline MHPR were similar in both CKD and non-CKD patients as compared with all patients. By contrast, in the fourth quartile, CKD patients with high baseline MHPR had a significantly greater reduction in MHSBP by 6.6 mm Hg compared with those with low baseline MHPR (*P*=.0084). Similarly, non-CKD patients had a significantly greater reduction in MHSBP by 2.2 mm Hg than those with low baseline MHPR (*P*=.0254). A similar pattern of reduction was also observed when the relation was analyzed by percentage reductions from baseline MHSBP (CKD Q4, *P*=.0123; non-CKD Q4, *P*=.0398). Differences of the changes in MHSBP between high and low MHPR were significantly modified by CKD and non-CKD patients (*P*=.0004).

## Discussion

In this study, a greater reduction in MHPR was observed in essential hypertensive patients with higher baseline MHSBP (≥165 mm Hg) after olmesartan-based treatment. Furthermore, a greater BP-lowering effect was noted in patients with high baseline MHPR (≥70 bpm) who had increased sympathetic nerve activity than those with low baseline MHPR (<70 bpm). This tendency was more evident in patients who had concomitant CKD. To our knowledge, this is the first study that reported the enhanced antihypertensive effect of olmesartan associated with sympathetic nerve activity in the clinical setting.

A significant reduction in PR was observed not only in patients with high baseline MHPR but also in those with a higher baseline BP who had a greater reduction in PR. Whereas sympathetic overdrive causes the development of hypertension, sympathetic nerve activity is also more potentiated according to the severity of hypertension.^19^ Although in this study we did not measure muscle sympathetic nerve activity (MSNA), which is a useful indicator of sympathetic nerve activity, an association between heart rate and MSNA has been reported.^7^ Thus, we consider that patients included in this study who had elevated BP and PR are highly likely to have increased sympathetic nerve activity. Sustained elevation of sympathetic nerve activity leads to increased cerebral and cardiovascular risk including cerebral hemorrhage and myocardial infarction.^20^ Antihypertensive drugs that suppress sympathetic nerve activity may, therefore, be effective for prevention of cardiovascular events. Generally, antihypertensive agents, except for β-blockers and some calcium channel blockers,^21^,^22^ have no effects on heart rate or even have a slightly adverse effect.

On the other hand, several studies using MSNA have shown that renin-angiotensin system inhibitors suppress sympathetic nerve activity without affecting baroreceptor sensitivity.^24^–^28^ However, few studies have reported the inhibitory effects of olmesartan on sympathetic nerve activity. Here, we provide new evidence for the effects of olmesartan in patients with increased sympathetic nerve activity. In a survey of the Japanese general population, an increase in morning heart rate by 5 bpm resulted in an increase in cardiovascular disease mortality by 17%.^17^ Therefore, a reduction in MHPR observed in this study may largely contribute to prevention of future cardiovascular events.

In the present study of patients with markedly high MHSBP, a subgroup with high baseline MHPR had a greater reduction in MHSBP by 3.2 mm Hg than those with low baseline MHPR. This tendency was more evident in CKD patients with a difference in BP reduction by 6.6 mm Hg. The reason for a greater BP reduction obtained in CKD patients with high baseline BP and PR may be because olmesartan not only inhibited the actions of Ang II but also suppressed the elevation of sympathetic nerve activity. CKD patients are known to have increased sympathetic nerve activity from the early stages,^12^ which is apparently caused by local ischemia of renal tissues.^29^ Renal ischemia induces activation of the afferent renal nerves, increases production of Ang II in the circulating blood, potentiates the activity of rostral ventrolateral medulla neurons, a sympathetic nervous center, and eventually increases sympathetic nerve activity to peripheral organs.^13^ Moreover, Ang II has a direct effect on the brain, leading to increased sympathetic nerve activity.^30^,^31^ In this way, the renin-angiotensin system and sympathetic nerve system activate each other, resulting in a vicious circle.^33^,^34^ ARBs suppress sympathetic nerve activity. A significant decrease in MSNA was observed in CKD patients who received treatment with an oral ARB.^25^,^26^ Thus, the effects of olmesartan on sympathetic nerve activity may be mediated through inhibition of Ang II in the brain and suppression of renal afferent nerve activity due to improvement of renal ischemia.

Our recent analysis of Jichi Medical University ABPM Study Wave 1 has shown that in hypertensive patients with CKD who also have elevated blood norepinephrine concentration, risk of stroke increases in a synergistic manner.^36^ Furthermore, in CKD patients, increased sympathetic nerve activity as shown by heart rate is associated with increased risks of developing end-stage renal disease or total mortality.^37^–^38^ Therefore, olmesartan may be particularly effective in the treatment of hypertensive patients who have concomitant CKD because of its effects on sympathetic nerve activity. In fact, a difference in BP reduction by 3 mm Hg has been reported to produce a reduction of cardiovascular events by 15%.^39^ Thus, olmesartan may be most beneficial for patients with elevated BP and PR, particularly when they have concomitant CKD. However, further prospective studies should be conducted to determine its effects in preventing events.

## Study Limitations

This study has several limitations. Firstly, this is a post-hoc analysis of an observational study with no control arm. Secondly, PR used in the present study, as an indicator of sympathetic nerve activity, is not a direct measurement. Nevertheless, there is clearly a close association between heart rate and renal sympathetic nerve activity, as shown by a marked decrease in heart rate following renal denervation.^40^ The results of the present study showing the antihypertensive effects of olmesartan using PR in the real-world setting are considered important regarding the possibility of an application in clinical practice.

## Conclusions

Olmesartan yielded statistically and clinically significant BP-lowering effects in patients with untreated essential hypertension with MHSBP ≥165 mm Hg in patients with high baseline MHPR compared with those with low baseline MHPR. Furthermore, in a subgroup of patients with CKD, an even greater reduction of MHSBP was noted in patients with high baseline MHPR than those with low baseline MHPR.
